# Semantical Visual Information Facilitates Odor Imagery: A Combined Neurophysiological and Psychometrical Approach

**DOI:** 10.1002/brb3.70835

**Published:** 2025-09-02

**Authors:** Luca Fantin, Hadrien Ceyte, Cécile Rumeau, Guillaume Drouot, Gabriela Hossu

**Affiliations:** ^1^ Université De Lorraine, Inserm, IADI Nancy France; ^2^ Normandie Université UNICAEN, ENSICAEN, CNRS, GREYC Caen France; ^3^ Aix Marseille Université, CNRS, ISM Marseille France; ^4^ Université De Lorraine, DevAH Nancy France; ^5^ CHRU‐Nancy, Université de Lorraine, Service ORL Nancy France; ^6^ CHRU‐Nancy, Inserm, Université de Lorraine, CIC, Innovation Technologique Nancy France

**Keywords:** crossmodal association, fMRI, odor imagery, olfaction

## Abstract

**Introduction:**

Odor imagery (OI), or the ability to mentally simulate the presence of a smell, is a difficult cognitive function and is therefore misunderstood in terms of its neural underpinnings. In particular, the diverging results obtained in neuroimaging studies could be explained in part by the characteristics of the visual cues used to trigger this task. In this study, we investigated this question by comparing the effects of plain color patches, pictures, and words during OI using neurophysiological and psychometrical measurements.

**Methods:**

Thirty healthy right‐handed participants performed an OI task during a functional magnetic resonance imaging exam. They were instructed to focus on the odors evoked by different types of visual cues. Two 595s functional runs were performed using a block design in which seven individual plain colors, pictures, and odor labels were used as visual triggers for OI. After each functional run, behavioral ratings of success/failure of OI as well as subjective pleasantness and intensity of odor images were collected.

**Results:**

The OI task induced activity not only in regions of the olfactory network but also in non‐olfactory brain regions. Across visual conditions, we observed activity in the orbitofrontal cortex, insula, supplementary motor area, and dorsolateral prefrontal cortex. Strikingly, no significant activity was found in the piriform cortex despite overall success in the OI task according to behavioral ratings (83% to 93% depending on the condition).

**Conclusions:**

Our findings suggest that OI can occur without significant involvement of the primary olfactory regions, contradicting most of the literature on this topic. Only little behavioral and neurophysiological effects of visual conditions were revealed in our sample of good odor imagers, although semantical cues seem to require fewer cognitive resources to generate odor images. This assumption remains to be confirmed using specific tools for the measurement of cognitive load.

## Graphical abstract

We investigated the influence of characteristics of visual cues provided during an odor imagery task on brain activity in fMRI. Several olfactory and non‐olfactory brain regions were activated, but not the piriform cortex. Neurophysiological and psychometrical comparisons between visual cues suggest slightly reduced cognitive load in presence of semantical information.

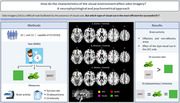

## 1 Introduction

In everyday life, many people report having spontaneous sensory experiences without corresponding external stimulation. Perhaps as a child when struggling to rest at night, you liked to picture sheep jumping over a fence until you fell asleep. Or maybe nowadays when you read a text message, you are able to hear the voice of your friend or significant other. These phenomena are forms of sensory imagery, which can be described as the mental simulation of sensory perceptions in the absence of corresponding physical stimulation (Andrade et al. 2012; Stevenson and Case 2005). Over the years, the scientific literature has focused on the comprehension of sensory imagery and its potential fields of application. Among its various forms, odor imagery (OI) constitutes a special case. Though OI has been witnessed for over a century (Betts 1910), the existence of an ability to form mental odor images has been a subject of debate (Engen 1991). This discussion was mainly due to the greater difficulty of OI compared to sensory imagery in other modalities, particularly vision or audition (Andrade et al. 2014; Lawless 1997; Schifferstein 2009). Today the scientific community has reached a consensus on the existence of OI, but because of its late acceptance, this cognitive function is still somewhat misunderstood, and its mechanisms are still debated. Therefore, several pieces of work in the past 20 years have explored the neural underpinnings of OI using neuroimaging. Findings have usually suggested that OI shares neural networks with odor perception, in a similar way to visual or tactile imagery (Olivetti Belardinelli et al. 2009). Performing an OI task has indeed been shown to induce activity in the piriform cortex (PC) as well as associative regions of the “extended olfactory network” (Gottfried 2010): the insula, the orbitofrontal cortex (OFC), and the hippocampus (Bensafi et al. 2007; Djordjevic et al. 2005; Flohr et al. 2014; Plailly et al. 2012). More recent findings (Leclerc et al. 2019) have highlighted the involvement of several brain regions associated with multimodal processing: the supplementary motor area (SMA), angular gyrus (AG), and inferior parietal lobule (IPS).

However, some results obtained from neuroimaging studies of OI remain contradictory. In particular, the precise role of the PC is still discussed (Royet et al. 2013). We believe that this may be caused by methodological variability, complicating the global interpretation of neurophysiological analyses of OI. Two main factors can be identified as potential sources of this variability. First, the ability to form mental odor images (OI ability) is an important source of bias, as it is known to be subject to wide interindividual variability. In this context, specific tools to measure OI ability have been developed, one of the most notable being the Vividness of Olfactory Imagery Questionnaire (VOIQ–Gilbert et al. 1998), which has also been translated and used in French and German populations (Fantin et al. 2020, 2022; Flohr et al. 2014; Kollndorfer et al. 2015). The use of the VOIQ and its translations has both confirmed the existence of good and bad odor imagers and helped identify or confirm some factors explaining OI ability. For example, expertise (Fantin et al. 2020; Gilbert et al. 1998) and age (Fantin et al. 2022) are positively related to OI ability, whereas olfactory loss and its severity (Fantin et al. 2020; Flohr et al. 2014; Kollndorfer et al. 2015) negatively affect OI ability. From a neurophysiological point of view, it has been shown that brain activity during an OI task differs between patients with olfactory loss and healthy non‐expert controls (Flohr et al. 2014). Although controls showed superior activation in the hippocampus and insula, patients with olfactory loss had increased activation of the dorsolateral prefrontal cortex (DLPFC), precuneus, and cerebellum. On the other end of the continuum, fragrance experts who are known for their excellent OI ability (Gilbert et al. 1998) have also been shown to differ from non‐experts (Plailly et al. 2012). Surprisingly, negative correlations were found between the amount of expertise and neural activation in several brain regions, particularly the PC, OFC, and hippocampus. This effect was discussed by the authors as a form of “*functional reorganization*” related to the increasing ease of the OI task in this expert population, owing to regular exposure to scented products and OI practice. Finally, within the non‐expert population, it has been demonstrated that patterns of activity in the PC differ between good and bad odor imagers when the pleasantness of OI is manipulated (Bensafi et al. 2007). Together, these findings highlight that OI ability is an individual characteristic that must be taken into account when interpreting results obtained from neuroimaging studies of this cognitive function.

Second, some variance in data can arise from the methodology used to help evoke odors during neuroimaging (functional MRI or PET) paradigms. To our knowledge, studies have used either auditory or visual cues to this end. The present work will focus on the latter, as numerous works have reported enhancement of performance in olfactory tasks in the presence of adequate visual input (Demattè et al. 2009; Gottfried and Dolan 2003; Zellner et al. 1991) on the basis of strong innate or acquired visuo‐olfactory crossmodal associations (see Spence 2011 for a review). We can therefore assume that visual cues during OI are relevant for facilitating performance in this challenging cognitive task. However, multiple types of visual cues have been used to explore the neurophysiological and behavioral responses to OI (most commonly odor labels, pictures, and plain colors), thereby leading to the question of which one to choose in experimental setups. Some findings suggest that the differences in the characteristics of visual cues may constitute a confounding effect on brain activity during visually evoked OI. Indeed, González et al. (2006) showed significant activation of the PC during the passive reading of words with strong olfactory connotations. This suggests that the primary olfactory cortex could be involved in top‐down cognitive processes such as the anticipation of a smell (Royet et al. 2013) or visuo‐olfactory crossmodal associations (Gottfried et al. 2002). Whether this effect is specific to odor labels remains unclear, but it does raise the question of the extent to which the characteristics of visual cues (complexity, presence of semantics, and presence of color) during OI can influence neural activity. In this context, a recent study (Hossu et al. 2024) used fMRI to compare brain activity during OI using pictures and non‐figurative colored arrangements (Jacquot et al. 2016) in order to determine the effect of figurativeness in complex and colored visual cues during OI. The results of this study revealed that in both conditions, OI induced activation in the insula and hippocampus but also in the SMA. However, no activation was revealed in the PC. The comparison between both conditions revealed little effect of the type of visual cues, with a significant difference only in the SMA, a frontal region involved in multisensory integration (Leclerc et al. 2019) and in cognitive load during OI (Han et al. 2022). At first sight, these results could suggest that the PC is not necessarily involved during OI in non‐expert healthy individuals. However, in the absence of any behavioral data (control of OI ability and subjective ratings of odor images during the MRI session), the obtained results are difficult to interpret. It remains unclear whether participants managed to successfully form mental odor images and, if so, to what extent. This work nevertheless suggests comparing the use of multiple types of visual stimuli during OI to further understand the effects of visual conditions on brain activity during this cognitive task.

The aim of this prospective, exploratory, single‐center study was to compare, using neurophysiological and psychometrical measurements, the use of different types of visual cues (plain color patches, pictures, and words) during an OI task in healthy good odor imagers.

## 2 Materials and Methods

### 2.1 Participants

Thirty right‐handed participants (16 women, 14 men) were included in this study. All participants presented a good OI ability, as expressed by the fVOIQ (Fantin et al. 2020). The fVOIQ is a 16‐item questionnaire in which participants are asked to imagine 16 smells divided into 4 everyday life situations. The vividness of odor images is reported on a scale ranging from 1 (perfectly realistic and as vivid as the actual odor) to 5 (no odor at all; I only “know” that I am thinking of the odor). Only participants who obtained a mean score below 2.5/5 were included in the study. This cutoff value was based on pretests during which healthy volunteers who obtained various fVOIQ scores were asked to perform OI evoked by visual cues other than those used in the present study. These pre‐tests helped determine that all volunteers with an fVOIQ score under 2.5 were able to very frequently evoke odor images (mean success rate >85%). The exclusion criteria were any disorder affecting their sense of smell, uncorrected visual impairment, or contraindication to MRI. The data of five participants (four women, one man) were discarded from the analyses because of excessive movement (>3 mm or 3°) during the fMRI runs. The statistical analyses were therefore performed on the 25 remaining participants. Details on this sample are provided in Table 1. The study received ethical approval from the local ethics committee (“CANOE” study; ClinicalTrials.gov Identifier: NCT05308433). Oral and written informed consent was obtained from each participant before inclusion.

**TABLE 1:** Descriptive characteristics of the studied sample after data exclusion for five participants due to excessive motion.

Characteristics	*N* = 25
Gender	
Man	15 [60%]
Woman	10 [40%]
Age	22.6 (3.5) | 19–33
fVOIQ score	1.92 (0.34) | 1.31–2.44

*Note: N* [%]; Mean (SD) | Min‐Max.

### 2.2 Experimental Procedure

All participants were first familiarized with the OI task they would perform during the MRI session. During this phase, participants were all successively presented with a plain orange (RGB [244 124 32]), a picture of lemons, and the word “chocolate” in French. The visual cues were presented in a random order. Participants were instructed to focus on the odor evoked by each of the visual cues and to notify whether they were able to generate an odor image. They were informed that they were free to imagine any odor they considered evoked by the visual cues and that there was no correct answer. When participants had no further questions, the MRI session could begin.

**FIGURE 1:**
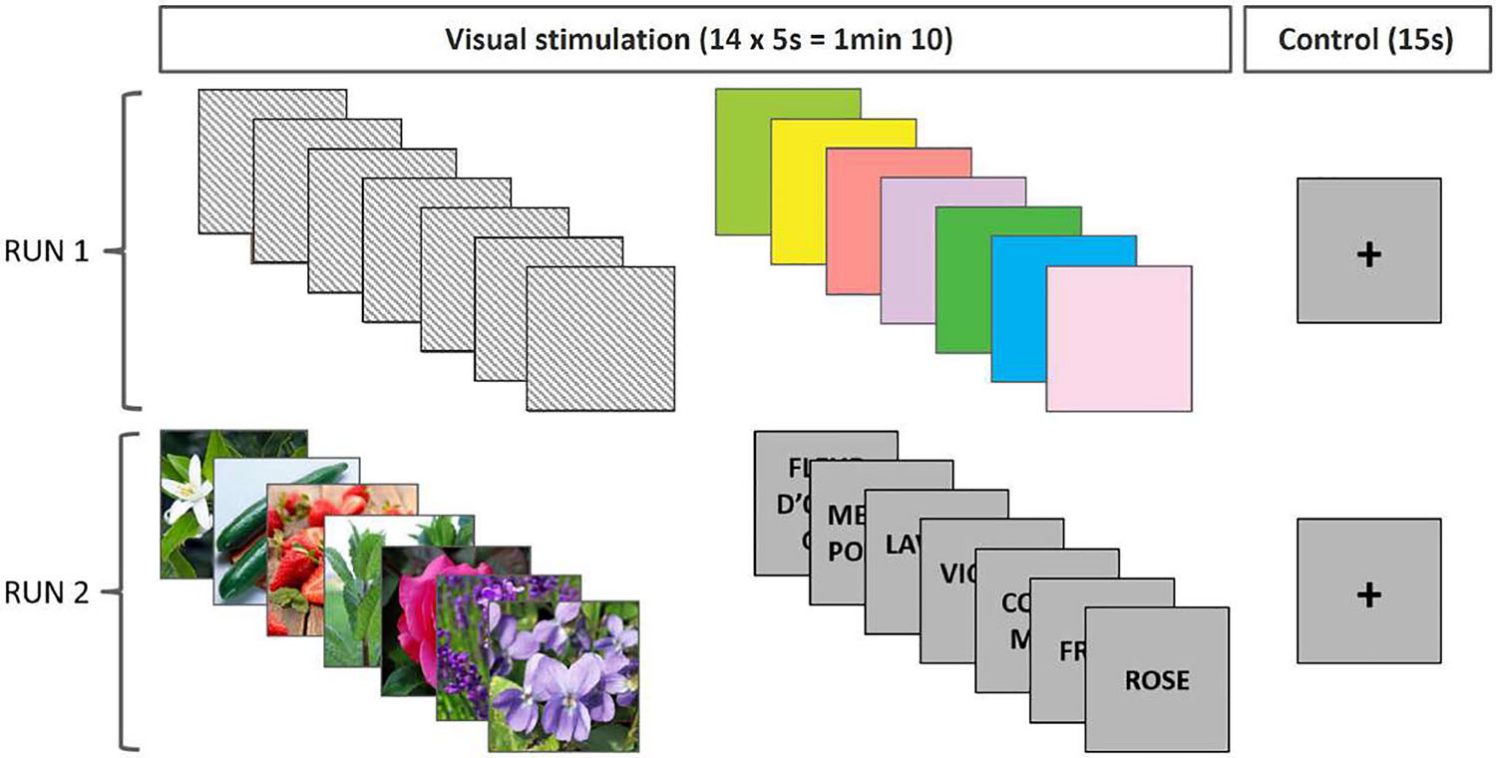
Paradigm used during both functional runs. One cycle for each run is depicted. The information regarding the first type of visual cue used in the first functional run has been removed for confidentiality purposes.

The OI fMRI paradigm consisted of two consecutive runs (Figure 1). In each run, a block design setup was used, comprising seven 85‐s cycles, for a total duration of 595 s (9 min 55 s). A cycle consisted of two 35‐s stimulation periods, each composed of seven different stimuli presented for 5 s, and a control period during which a gray screen with a black fixation cross was presented for 15 s. In total, the paradigm comprised four visual conditions. The first functional run consisted of seven abstract, complex, colored visual cues protected by intellectual property (patented technology protected by a material transfer agreement) and seven plain color patches of the colors green [78 183 72] and [158 201 60], yellow [247 236 31], pink [255 146 141] and [249 217 232], purple [221 194 221], and blue [1 176 241]. The second functional run consisted of seven pictures representing rose, violet, lavender, cucumber, strawberry, orange blossom, and peppermint; then the French labels of these objects, written in black on a gray background. Given considerations related to intellectual property, the visual cues based on a patented technology cannot be presented in our study. Therefore, the results related to this condition will not be reported or discussed. However, we would like to draw the readers’ attention to the fact that both functional runs were identical in their design, which is an important methodological consideration for fMRI data processing. The three remaining types of visual cues were chosen on the basis of two factors. First, they are the most commonly used methods in neurophysiological and behavioral studies of OI. Second, they possess complementary characteristics that are of interest for our question. Specifically, they are either complex, colored and semantical (pictures), non‐colored and semantical (odor labels), uniform, or colored and abstract (plain color patches). Moreover, the choice of the individual odorous objects depicted was validated in a previous study of OI neuroimaging (Hossu et al. 2024).

During the OI task, participants were instructed to focus on the odors evoked by each visual cue that was presented. As each unique cue was presented 7 times, one functional run consisted of 98 repetitions of this visuo‐olfactory evocation task. During the control period (fixation cross), participants were asked to simply look at the cross. The order of presentation of the visual cues within each stimulation phase was randomized between cycles. However, the structure of the paradigm (order of the stimulation phases and order of runs) was identical for all participants: the seven patented visual cues and seven plain color patches, followed by the seven pictures and seven words. This ensured that the visual interpretation of the plain color patches was not influenced by prior knowledge of the depicted odor objects. Participants were explicitly told that they did not have to identify the depicted odors and that they were free to imagine any odor they thought corresponded to the visual stimuli.

After each functional run, all 14 visual cues were projected again. For each individual visual cue, participants were asked to focus on the evoked odor and to rate the pleasantness and intensity of the generated odor image separately. To this end, they were equipped with a fourth‐level response box in their right hand (fORP response box, four button inline, Current Designs, USA) inside the MRI scanner. A tactile cue was placed on one end of the box, indicating the highest level of pleasantness/intensity. If one visual cue did not allow for the creation of any odor image, participants were instructed not to press any button on the response box, and the next visual cue was presented after 10 s.

### 2.3 MRI Data Acquisition

The visual cues were forward‐projected on a translucent screen placed inside the MRI room, using a calibrated projector (PT‐AE500E, Panasonic, Kadoma, Osaka, Japan). A mirror was attached to the head coil, allowing participants to see the screen from inside the MRI scanner. All visual cues were projected using ePrime (v3.0, Psychology Software Tools Inc., Pittsburgh, PA, USA) and synchronized with the MRI. ePrime also allowed for the collection of the subjective ratings of the odor images using the response box. Vision correction was performed using non‐magnetic glasses, which can vary in the range of −5 to +5 with 0.5 diopter steps. Whole brain functional MR images were acquired using a 3‐Tesla magnet (Magnetom Prisma, Siemens AG, Germany) equipped with a 64‐channel receive‐only head coil. For all participants, high‐resolution anatomical images were acquired using a 3D turbo flash (TFL) sequence with the following parameters: repetition time (TR) = 2200 ms; echo time (TE) = 2.91 ms; flip angle 8°; in plane resolution 0.94 × 0.94 mm^2^; slice thickness 1 mm. One hundred and sixty volumes parallel to the anterior–posterior commissural line covered the whole brain, extending from the vertex to the inferior parts of the cerebellum. For each functional run, 238 volumes of BOLD gradient‐echo echo‐planar images (GE‐EPI) were obtained with the following imaging parameters: TR = 2500 ms, TE = 30 ms, 40 axial slices, slice thickness 3 mm, slices order: interleaved, in plane resolution 2 × 2 mm^2^, 0.75 mm spacing between slices, and flip angle 77°.

### 2.4 Data Processing and Analysis

MRI data preprocessing and analysis were performed using SPM12 software (Statistical Parametric Mapping, Version 12, Revision 7219, Wellcome Department of Cognitive Neurology, London, UK). The standard preprocessing pipeline consisted of motion correction, spatial normalization, and spatial smoothing using a 6 × 6 × 6 mm^3^ full‐width half‐maximum Gaussian kernel. The difference between stimulation (plain color patches, pictures, or words) and the respective control conditions (fixation cross) was statistically evaluated in the form of a block design. The first‐level statistical analysis consisted of a general linear model including realignment parameters and the subtraction of BOLD signal during the control condition to that during the stimulation condition ([Colors > Control]; [Pictures > Control]; [Words > Control]). This process was performed for each visual condition separately. The weight of the stimulation conditions was set to 1, and the weight of the control periods was set to −1 (generically named [Stimulation > Control] contrast). The stimulation and control periods were modeled as boxcar regressors with durations corresponding to the full durations of the corresponding conditions for each cycle (i.e., 35 s). The six motion parameters (3‐dimensional translation and rotation) were added to the model as nuisance regressors. A 128‐s high‐pass filter was used to remove non‐physiological slow signal shifts. Contrasted parameter estimates images were then calculated at the group level using a 1‐sample *t*‐test.

Second‐level analyses also included a repeated measures ANOVA (degrees of freedom = {3.72}) implemented to determine whether the visual conditions of the OI task could influence the obtained BOLD signal. If a main effect of type of visual cue was revealed by the ANOVA in a selected region of interest (ROI), bilateral paired comparisons were performed between [Stimulation > Control] contrasts in this ROI. The selected ROIs were the PC (Seubert et al. 2013) and the “extended olfactory network” as described by Gottfried (2010), which includes the amygdala, OFC, insula, and hippocampus (McNorgan 2012). The thalamus, SMA (Leclerc et al. 2019), middle frontal gyrus, and superior frontal gyrus (Flohr et al. 2014) were also included. ROI masks were generated from the “neuromorphometrics” template (MNI space) introduced in SPM and were then registered to the functional space. The PC was defined from a mask obtained from the authors of Seubert et al. (2013). For first‐ and second‐level ROI analyses, the threshold was set to *p* < 0.001 at the voxel level, uncorrected for family‐wise errors and multiple comparisons. A minimal number of 10 voxels per cluster was defined. For exploratory purposes, pairwise comparisons between visual conditions at the second level were also performed using whole‐brain analyses. For these tests, the significance threshold was set to *p* < 0.001 (uncorrected), and the minimal number of 30 voxels per cluster was set in order to reduce the false discovery rate. All graphical representations of brain activity were generated under MRIcron (v.1.0.20190902) using the ch2bet template.

Behavioral ratings were analyzed to ensure that participants had in fact managed to generate mental odor images and to compare the quality and quantity of odor images under each visual condition. To this end, the pleasantness and intensity ratings were encoded into scores ranging from 1 (lowest pleasantness/intensity) to 4. For each participant and each type of visual cue, 3 scores were obtained: the number of generated odor images (0 to 7), the mean pleasantness of odor images (1 to 4), and the mean intensity of odor images (1 to 4). For each of these 3 scores, the effect of the type of visual cue was tested. As the scores did not follow a normal distribution according to Shapiro–Wilk tests, Friedman tests were used. In the case of a main effect, pairwise comparisons were performed using pairwise Wilcoxon tests, with Benjamini–Hochberg *p* value adjustment method. Quantitative data are expressed as means and standard deviations (SD) or medians (Med) and inter‐quartile ranges (IQR). Qualitative data are expressed as frequencies. All statistical analyses of the behavioral data were performed under RStudio (version 2024.09.1 + 394). The rstatix package was used to perform Friedman and Wilcoxon tests and to determine effect sizes. All statistical thresholds for behavioral data during repeated measures analyses were corrected according to the number of multiple comparisons.

## 3 Results

### 3.1 Behavioral Data

Results revealed a significant effect of the type of visual cue on the number of evoked odors (*F*(3) = 12.2; *p* = 0.007; Friedman effect size = 0.16). Paired comparisons revealed that plain color patches (Med = 6; IQR = 1) evoked significantly fewer odors than words did (Med = 7; IQR = 1; *p* = 0.023; Wilcoxon effect size = 0.53). However, there were no significant effects of the type of visual cue on the mean pleasantness or intensity of the evoked images (*F*(3) = 3.15 and *F*(3) = 4.46, respectively; *p* > 0.05). Detailed values of the mean pleasantness and intensity scores are provided in Table 2.

**TABLE 2:** Number, pleasantness, and intensity of evoked odor images for each type of visual cue during the odor imagery (OI) task.

Variable	Plain colors	Pictures	Words	*p* value^a^
Number of evoked odors	6 (1)	7 (1)	7 (1)	**0.007**
Overall rate of successful odor imagery	82.9%	90.3%	93.1%	
Mean pleasantness	3.2 (0.6)	3.3 (0.6)	3.3 (0.7)	0.369
Mean intensity	2.8 (0.7)	2.9 (0.8)	3.0 (1.0)	0.216

*Note*: Median (IQR).

^a^ Friedman test.

In bold signficant p value i.e. p < 0.05

### 3.2 fMRI Data

**FIGURE 2:**
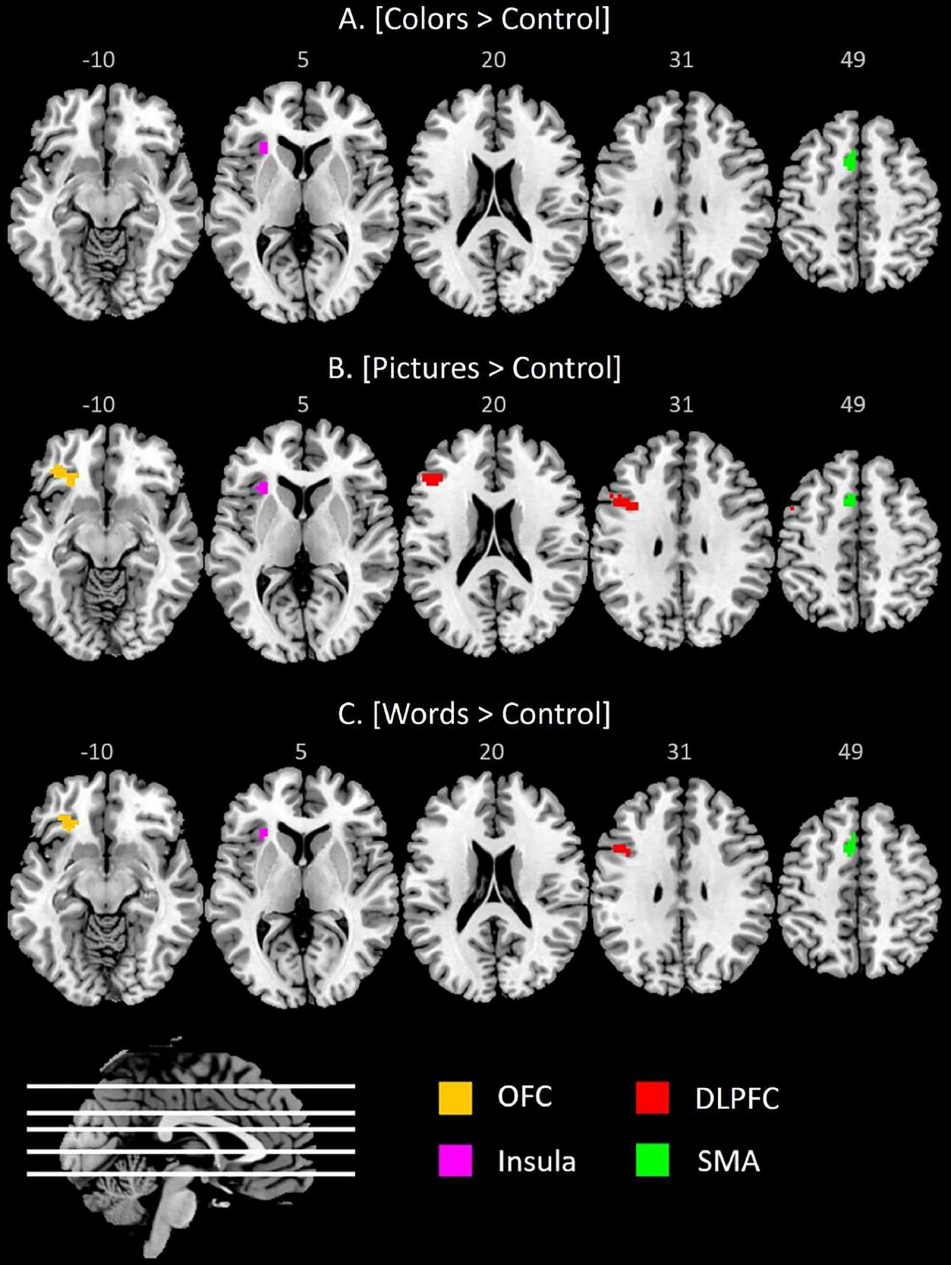
Brain activity for each [Stimulation > Control] contrast within the selected regions of interest. Statistical threshold was set at *p* < 0.001 (uncorrected), *k* = 10. DLPFC, dorsolateral prefrontal cortex; OFC, orbitofrontal cortex; SMA, supplementary motor area.

All three [Stimulation > Control] contrasts induced significant activation in the left insula and the SMA. Additionally, in the case of the [Pictures > Control] and [Words > Control] contrasts, we observed significant unilateral activation in the left OFC and middle frontal gyrus of the DLPFC. The clusters are depicted in Figure 2, and their details (size, location, and intensity) are provided in Table 3.

**TABLE 3:** Region of interest (ROI) results for the [Stimulation > Control] contrasts.

Contrast	ROI	Hemisphere	Size	*x*	*Y*	*z*	Peak *t*	
Plain colors > Control	Insula	L	17	−27	20	11	4.21	
	SMA	L	48	−9	14	47	5.73
Pictures > Control	OFC	L	67	−21	23	−16	5.67	
	Insula	L	40	−27	23	8	4.16	
	SMA	L	51	−6	8	56	4.85	
	Middle frontal gyrus	L	119	−36	32	17	5.38	
Words > Control	OFC	L	24	−27	32	−10	4.62	
	Insula	L	30	−30	23	8	4.51	
	SMA	L	40	−6	11	53	8.71	
	Middle frontal gyrus	L	30	−45	11	32	5.23	

*Note*: Only activated clusters within the investigated regions of interest are displayed (*p* < 0.001, uncorrected, *k* = 10). 3‐dimensional coordinates of the local maxima as well as peak *t*‐scores are provided. Size is expressed in voxels.

**^Abbreviations:^ :**L, left; OFC, orbitofrontal cortex; ROI, region of interest; SMA, supplementary motor area.

**FIGURE 3:**
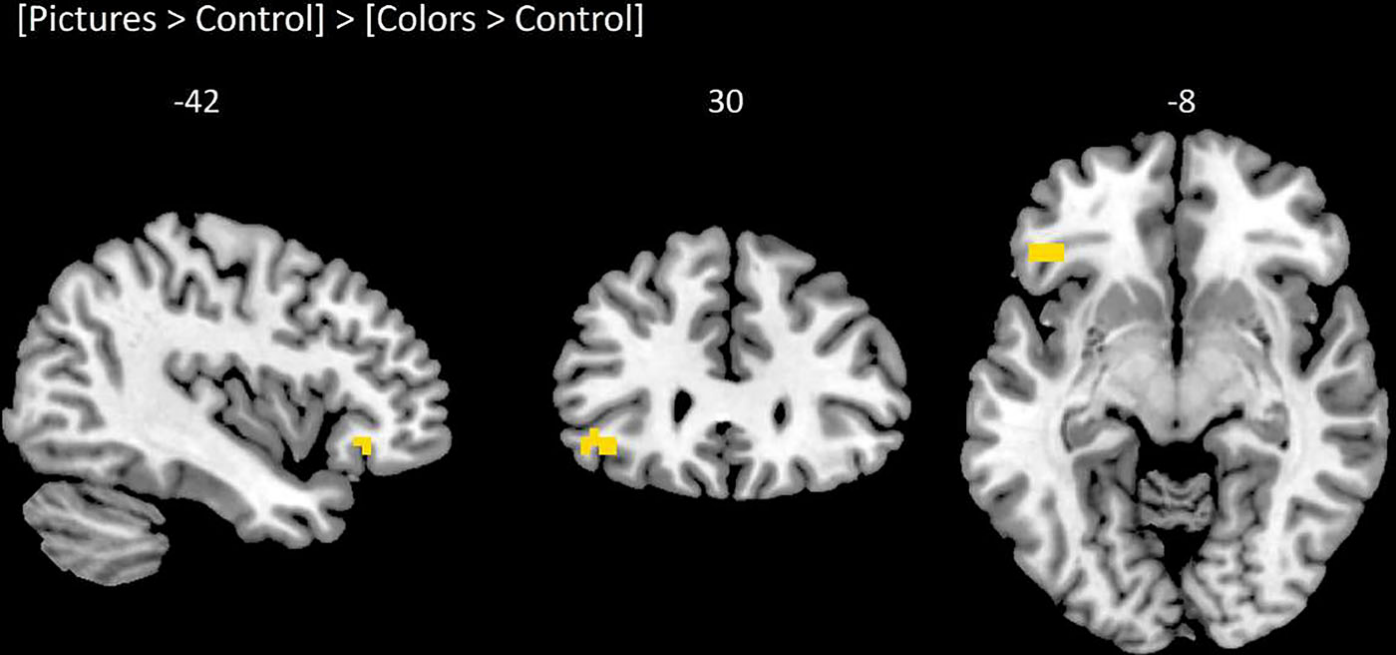
Brain activity in the left OFC for the [Pictures > Control] > [Colors > Control] contrast. Statistical threshold was set at *p* < 0.001 (uncorrected), *k* = 10.

The comparison of [Stimulation > Control] contrasts using a repeated‐measures ANOVA revealed a significant effect of stimulation condition only in the left OFC. Pairwise comparisons showed greater activity in the left OFC for [Pictures > Control] than for [Colors > Control] (Figure 3). The details of this cluster of activation are provided in Table 4.

**TABLE 4:** Details on the cluster of activity in the orbitofrontal cortex (OFC) extracted from paired comparisons within regions of interest (*p* < 0.001, uncorrected, *k* = 10).

Contrast	ROI	Hemisphere	Size	*x*	*Y*	*z*	Peak *t*
[Pictures > Control] > [Plain colors > Control]	OFC	L	14	−48	28	−10	4.26

*Note*: 3‐dimensional coordinates of the local maxima as well as peak *t*‐scores are provided. Size is expressed in voxels.

Abbreviation: L, left; OFC, orbitofrontal cortex; ROI, region of interest.

Further comparison of the visual conditions via whole‐brain analyses revealed several significantly activated clusters for each pairwise comparison. Although most of these clusters were located in secondary visual areas, our results did reveal interesting differences in the fusiform gyrus and the AG. The detailed results are provided in Table S1.

## 4 Discussion

The aim of this study was to compare, using neurophysiological and behavioral measurements, the use of different types of visual cues during an OI task in healthy good odor imagers. Although four conditions were part of the initial paradigm, only plain color patches, pictures, and words were compared and will be discussed in this section. Across visual conditions, we observed activity in the left OFC, left insula, SMA, and DLPFC. Overall, our results corroborate the presence of a mainly left‐lateralized activation network often reported during OI for right‐handed subjects. Although left lateralization of brain activity has often been associated with pleasantness, findings on this matter are less consistent in the case of OI (Bensafi et al. 2007). Independently of hedonic considerations, our findings seem to confirm that OI primarily recruits the left hemisphere of the brain. In all tested visual conditions, OI induced significant activity in the left insula and the SMA, both of which are known for their role in multisensory integration (Leclerc et al. 2019; Uddin et al. 2014, 2017), thus corroborating the idea of a multimodal nature of OI (Nanay 2018). The insula is also considered a secondary olfactory brain region (Gottfried 2010), although its specific function in the processing of olfactory stimuli remains unclear (Roy‐Côté et al. 2021). Additionally, the use of both pictures and words induced significant clusters of activation in the left OFC and left middle and superior frontal gyri (DLPFC). Although the OFC has many functions related to olfaction and visuo‐olfactory association (Gottfried and Dolan 2003; Österbauer et al. 2005), the DLPFC is rather involved in cognitive load (Cieslik et al. 2013; Whelan 2007). Altogether, our results partially reproduce prior findings, showing the engagement of both olfactory and non‐olfactory brain regions during OI.

One striking result was the absence of significant activation in the PC during all visual conditions of OI. This finding contradicts most of the existing literature, particularly during the use of odor labels (Bensafi et al. 2007; Djordjevic et al. 2005), and raises questions about the involvement of the primary olfactory cortex during OI. To our knowledge, similar observations have been reported in two previous studies: one involving healthy non‐experts (Hossu et al. 2024) and one involving fragrance experts (Plailly et al. 2012). In Hossu et al., it was suggested that the absence of significant activation in the PC could be attributed to the characteristics of the visual cues used in their fMRI paradigm, a hypothesis that was not verified using odor labels. Moreover, no behavioral ratings of odor image quality were collected, making it difficult to determine to what extent participants had succeeded in the OI task and how this factor could be related to the absence of significant PC activation. However, in Plailly et al. (2012), participants were trained students and professionals in perfumery, and successful OI was confirmed by behavioral ratings. Although, according to the authors, decreased PC activation may have been caused by a form of functional reorganization, we propose that this effect could also be of behavioral origin. Indeed, it is known that OI usually results in sniffing (Bensafi et al. 2003, 2005; Kleemann et al. 2008), and that the PC is activated in response to sniffing without smelling (Sobel et al. 1998). However, the action of sniffing during OI could rather be considered a strategy to enhance performance, either through the retrieval of odors in long term memory (Kosslyn 2003) or through reenactment of sensory motor feedback experienced during olfactory perception (Pylyshyn 2003). In a population of experts with above‐average OI ability, this strategy may not have been necessary for successful OI. In fact, in Plailly et al.’s study, participants were explicitly asked not to sniff, which did not affect their performance. Given that we specifically targeted good odor imagers using the fVOIQ, a similar effect may have been produced. Indeed, in the present study, the success or failure of OI was controlled by ratings of pleasantness and intensity of odor images evoked by each visual cue. Overall, participants reported successfully evoking odor images in most trials, with rates of success (90% to 93% in the pictures and words conditions) similar to those reported by Plailly et al.’s expert population (92% to 93%). It is therefore possible that our sample of good odor imagers did not need to use sniff to enhance their performance, explaining the absence of PC activation. Unfortunately, breathing was not monitored in our study but should become a standard measurement in future works seeking to understand OI. In particular, the investigation of sniffing patterns in good versus bad odor imagers seems relevant for further understanding how olfactomotor behavior influences OI‐induced brain activity. This question has previously been investigated (Bensafi et al. 2005); however, the samples were quite small (four vs. six participants), and groups were defined using a clustering technique among the recruited participants, rather than an a priori threshold determining a satisfactory level of OI ability. Further investigating the question of individual OI ability could therefore provide more answers on the potential variability of neural networks involved during this task, and how they are affected by sniffing behaviors. Future studies could include bad odor imagers, or participants with aphantasia, to understand the extent to which unsuccessful reenactment of sensory stimulation affects brain activity.

In this study, we directly compared the use of different types of visual cues during OI. Our behavioral data revealed only little differences between the plain colors, pictures, and words. Although the use of plain colors seemed to be less efficient in evoking smells, it is important to note that the OI task was globally well performed, with mean success rates ranging from 82.9% to 93.1%. From a neurophysiological perspective, our findings showed increased activity in the left OFC during the use of pictures compared with plain color patches. As it stands, this result is difficult to interpret in terms of the quality of OI given the absence of significant differences between both visual conditions regarding the amount, pleasantness, and intensity of odor images in our sample. However, the OFC has often been discussed for its primary role in visuo‐olfactory association (De Araujo et al. 2005; Gottfried and Dolan 2003; Österbauer et al. 2005). In particular, several studies have shown that the level of correspondence between simultaneous visual and olfactory stimulation (visuo‐olfactory congruency) can modulate activity in this region. Gottfried and Zelano (2011) have therefore discussed the great “importance of context and experience in formation of multisensory representations in human OFC.” In this conception, plain color patches are the type of visual cue offering the least context due to their simplicity and lack of semantics, whereas pictures provide the closest similarity to an ecological encounter with an odorant object. The increased activity in the OFC in the pictures condition may therefore be attributed to these characteristics of visual cues, which while they do not affect the quality of OI in good odor imagers, may influence the amount of attentional resources invested in this task. Indeed, according to several reports (Leclerc et al. 2019; Nanay 2018), stimuli are processed as multimodal during OI, implying that the creation of an odor image may require a form of identification of the visually depicted object. In the case of non‐semantical visual cues such as color patches, the reconstruction of semantical meaning could have increased the attentional demand during the OI task. However, it is important to note that no direct measure of cognitive load was performed in our study, which is why this interpretation is purely hypothetical. Future studies of OI could implement specific tools such as the NASA Task Load Index (NASA‐TLX–Hart and Staveland 1988) in order to explore this question. Additionally, given the supposed multimodality of OI (Leclerc et al. 2019; Nanay 2018) and the position of the OFC at the convergence of several sensory pathways (Rolls 2008), performing connectivity or multivariate pattern analyses, which are more network‐oriented and may be more adapted to multisensory integration matters, could be relevant. This approach may reveal some effects of the visual context that are not visible when each ROI is independently considered. Moreover, such analyses could provide more insight into the activation patterns of the PC, which is known to use distributed spatial patterns to encore odor identity.

Our exploratory whole‐brain analyses comparing visual cues revealed the potential involvement in OI of structures outside of our regions of interest. Although most of this activity was located in the occipital regions (which is logical given the changes in visual stimulation induced by each condition), we were also able to demonstrate cue‐dependent activity in the fusiform gyrus and the AG. The fusiform gyrus presented more activity in the pictures and colors conditions compared to the words condition. The involvement of this region in olfaction and multisensory integration is unclear in current studies, and its functions seem to be apparently more related to visual processing, namely, object recognition and color knowledge (Wang et al. 2013; Weiner and Zilles 2016). With respect to the AG, our results revealed increased activity in this region for the words and pictures conditions compared with the plain colors condition. Although this could be simply due to the alleged role of this region in the retrieval of semantical episodic memories and semantic processing (Bonnici et al. 2016; Seghier 2013), the AG is also known to be a cross‐modal hub. At the border between the temporal, parietal, and occipital lobes, it receives converging multisensory information (Hirst et al. 2021; Seghier 2013). This brain region could therefore become an interesting topic of focus for future studies on the neurophysiology of OI and sensory imagery.

### 4.1 Limitations and Perspectives

Our results must, however, be cautiously interpreted with respect to possible confounding factors. The first factor is the absence of quantitative measurements of our participants’ olfactory abilities. The present study relied on declaration alone to exclude any participants with smell disorders, and it has been shown that these disorders can negatively affect performance in OI tasks (Fantin et al. 2020; Kollndorfer et al. 2015). However, previous works have shown that individuals with olfactory disorders hardly ever obtain fVOIQ scores lower than 2.5/5 (Fantin et al. 2020). We believe that screening on the basis of OI performance (which was the subject of our main objective) and the declaration of participants was sufficient to characterize our participants as healthy. Moreover, we wished to limit any olfactory stimulation preceding the fMRI session. Performing a task such as the *Sniffin’ Sticks* test (Hummel et al. 2007) could have induced an initial state of olfactory fatigue in our participants, consequently affecting their performance during the OI task.

The second limitation of this study is the lack of consideration of potential gender‐dependent effects of OI on brain activity. It has indeed been discussed that in olfactory tasks, men and women perform differently, and some studies tend to demonstrate better performance in women. In the case of OI, it seems that gender does not affect performance in healthy normosmic individuals (Fantin et al. 2022). Moreover, our fVOIQ scores did not significantly differ between men and women in the present study. The remaining question is whether the implicit processing of visual cues during an OI task may induce differential neural activity. Although this specific question has not yet been addressed, sex‐specific effects have been demonstrated in multisensory integration brain regions (temporal areas, inferior frontal gyrus, and hypothalamus) during an olfactory task (Melero et al. 2019). If OI also involves multisensory integration processes as discussed, the brain regions involved in this task could similarly be affected. Unfortunately, in the present study we chose not to investigate the differences between men and women to preserve statistical power in an experimental design with multiple conditions. Yet, it offers a very interesting perspective for future studies wishing to better understand the mechanisms of OI, and perhaps sensory imagery broadly speaking.

The final possible bias is the order of the experimental conditions. The functional runs were purposely set to present the abstract visual cues first, neutralizing any potential influence of previous exposure to pictures and odor labels. However, this methodological choice raises concerns as to whether cognitive fatigue may have affected our results, given that pictures and words were systematically presented during the second run. This issue is difficult to resolve, but we would like to address it by raising several points. First, participants were healthy individuals and were explicitly recruited using the fVOIQ in order to ensure that they were capable of evoking smells well. Presumably, such a population may experience less fatigue, although it is likely that an effect of order still occurred to a certain extent. To limit this effect, all participants were given a short time to rest (approximately 5 min) between runs. Second, informal interviews were performed with participants at the end of the experimental sessions to discuss any of their impressions regarding the task. Although some did find the OI task difficult overall, no participant expressed the inability to concentrate or to perform OI during the second run. In fact, psychometrical measurements do suggest that the OI success rate increased in the presence of semantical cues, which were rated at the end of the session. To determine the extent to which repetition can affect the quality of OI and the neural underpinnings of this fatigue, future studies could consider using an event‐related design, with psychometrical ratings after each repetition.

## 5 Conclusion

This study corroborates the observations of OI involving olfactory and non‐olfactory brain regions when evoked by visual cues. However, no significant activation was observed in the PC despite reports of successful OI, suggesting that this brain region is not necessarily involved in this cognitive task but may rather be activated by behavioral strategies aimed at enhancing OI performance. Together, our data do not allow for a firm conclusion on differences between types of visual cues to facilitate OI, although the behavioral and neurophysiological comparisons between types of visual cues tend to suggest that semantical cues may be slightly preferable to plain color patches to reduce the attentional cost necessary to evoke odors. Nevertheless, further studies are still needed to determine to what extent cognitive load is affected by the visual conditions of OI.

## Author Contributions

**Luca Fantin**: conceptualization, methodology, investigation, validation, visualization, writing–review and editing, writing–original draft. **Hadrien Ceyte**: conceptualization, methodology, validation, supervision, writing–original draft, writing–review and editing. **Cécile Rumeau**: supervision, writing–original draft. **Guillaume Drouot**: project administration, writing–original draft. **Gabriela Hossu**: conceptualization, methodology, investigation, validation, supervision, writing–original draft, writing–review and editing.

## Peer Review

The peer review history for this article is available at https://publons.com/publon/10.1002/brb3.70835

## Supporting information




**Supplementary Table**: brb370835‐sup‐0001‐TableS1.docx

## Data Availability

The data that support the findings of this study are available on request from the corresponding author. The data are not publicly available due to privacy or ethical restrictions.
