# Supplementary material for: Semantical Visual Information Facilitates Odor Imagery: A Combined Neurophysiological and Psychometrical Approach
Source: Brain Behav. 2025 Sep 2;15(9):e70835. doi: 10.1002/brb3.70835 (PMC12405669; doi:10.1002/brb3.70835)
Supplement: Supplementary file 1 — Supplementary Table: brb370835‐sup‐0001‐TableS1.docx [file BRB3-15-e70835-s001.docx]

Supplementary Table 1. Results for the pairwise comparisons extracted from whole-brain analyses (p < 0.001, uncorrected, k = 30). 3-dimensional coordinates of the local maxima as well as peak t-scores are provided. Size is expressed in voxel. L: Left, R: Right. Displayed results do not include clusters located in the regions of interest (provided in the main text).

| Contrast | Region | Size | *x* | *y* | *z* | Peak *t* | |
| --- | --- | --- | --- | --- | --- | --- | --- |
| [Plain colors > Control] > [Pictures > Control] | Superior occipital gyrus (R) | 42 | 15 | -88 | 20 | 5.75 |  |
|  | Lingual gyrus (R) | 72 | 12 | -76 | -4 | 5.2 | |
| [Pictures > Control] > [Plain colors > Control] | Inferior occipital gyrus (R) | 1007 | 33 | -85 | -7 | 15.24 | |
|  | Inferior occipital gyrus (L) | 1404 | -24 | -91 | -7 | 12.19 | |
|  | Cerebellum (R) | 46 | 27 | -67 | 29 | 5.51 | |
|  | Gyrus rectus | 44 | 0 | -55 | 17 | 5.01 | |
|  | Angular Gyrus (L) | 30 | -42 | -49 | 26 | 4.49 | |
| [Plain colors > Control] > [Words > Control] | Fusiform gyrus (L) | 56 | -27 | -58 | -13 | 7.77 | |
|  | Lingual gyrus (R) | 43 | 12 | -100 | -11 | 5.46 | |
|  | Fusiform gyrus (R) | 102 | 27 | -52 | -10 | 5.07 | |
| [Words > Control] > [Plain colors > Control] | Inferior occipital gyrus (L) | 104 | -27 | -94 | -7 | 7.28 | |
|  | Inferior occipital gyrus (R) | 68 | 36 | -88 | -7 | 6.46 | |
|  | Angular gyrus (L) | 77 | -42 | -52 | 23 | 5.64 | |
|  | Inferior temporal gyrus (L) | 36 | -45 | -64 | -7 | 4.9 | |
| [Pictures > Control] > [Words > Control] | Fusiform gyrus (R) | 1582 | 27 | -85 | -1 | 11.39 | |
|  | Fusiform gyrus (L) | 1390 | -33 | -67 | -13 | 10.14 | |
| [Words > Control] > [Pictures > Control] | Cuneus (R) | 630 | 15 | -85 | 23 | 8.04 | |
